# Primer pairs, PCR conditions, and peptide nucleic acid clamps affect fungal diversity assessment from plant root tissues

**DOI:** 10.1080/21501203.2023.2301003

**Published:** 2024-02-04

**Authors:** Chloé Viotti, Michel Chalot, Peter G. Kennedy, François Maillard, Sylvain Santoni, Damien Blaudez, Coralie Bertheau

**Affiliations:** a CNRS, Chrono-environnement, Université de Franche-Comté, Montbéliard, France; b Faculté des Sciences et Technologies, Université de Lorraine, Nancy, France; c Department of Plant & Microbiology, University of Minnesota, St. Paul, MN, USA; d AGAP Institut, Univ Montpellier, CIRAD, INRAE, Institut Agro, Montpellier, France; e Université de Lorraine, CNRS, LIEC, Nancy, France

**Keywords:** ITS2, fungi, annealing temperature, metabarcoding, high-throughput sequencing, blocking primers, *Urtica dioica*

## Abstract

High-throughput sequencing has become a prominent tool to assess plant-associated microbial diversity. Still, some technical challenges remain in characterising these communities, notably due to plant and fungal DNA co-amplification. Fungal-specific primers, Peptide Nucleic Acid (PNA) clamps, or adjusting PCR conditions are approaches to limit plant DNA contamination. However, a systematic comparison of these factors and their interactions, which could limit plant DNA contamination in the study of plant mycobiota, is still lacking. Here, three primers targeting the ITS2 region were evaluated alone or in combination with PNA clamps both on nettle (*Urtica dioica*) root DNA and a mock community. PNA clamps did not improve the richness or diversity of the fungal communities but increased the number of fungal reads. Among the tested factors, the most significant was the primer pair. Specifically, the 5.8S-Fun/ITS4-Fun pair exhibited a higher OTU richness but fewer fungal reads. Our study demonstrates that the choice of primers is critical for limiting plant and fungal DNA co-amplification. PNA clamps increase the number of fungal reads when ITS2 is targeted but do not result in higher fungal diversity recovery at high sequencing depth. At lower read depths, PNA clamps might enhance microbial diversity quantification for primer pairs lacking fungal specificity.

## 1. Introduction

Fungi are ubiquitous microorganisms that play a number of critical ecological roles in ecosystems (Peay et al. 2016; Dighton 2018). Notably, many fungal species across diverse lineages interact with and grow in close association with cultivated and wild plants (Nguyen et al. 2016; Zeilinger et al. 2016). Fungi are frequently found both around and within plant tissues, particularly in roots, as part of the root microbiome (Trivedi et al. 2020), and are involved in both plant nutrient acquisition and resistance to pathogens (Smith and Read 2010; Waqas et al. 2015; Diagne et al. 2020). Thus, there has been a growing interest in characterising the diversity and composition of plant-associated fungal communities over recent decades (Trivedi et al. 2020; Bollmann-Giolai et al. 2022). High-throughput amplicon sequencing (HTS), also called metabarcoding, has emerged as a powerful tool for analysing complex microbial communities from environmental samples (Nilsson et al. 2019; Baldrian et al. 2021). Yet, HTS applied to fungal communities associated with plants is challenging, given that plant DNA can contaminate the sequencing results. For example, amplicons deriving from plant DNA have been found to be dominant in many metabarcoding studies exploring the plant mycobiota (Almario et al. 2017; Fabiańska et al. 2019). As plant-fungal DNA co-amplification can significantly reduce the sequencing depth, this might lead to an underestimation of the plant-associated fungal diversity (Mayer et al. 2021). New techniques such as host-associated microbiome PCR, aim to co-amplify microbial markers and host genes to determine microbial load and community composition by adjusting the host: microbe ratio (Lundberg et al. 2021).

The ribosomal internal transcribed spacer (ITS) region is considered the primary fungal DNA barcode (500–700 bases) (Schoch et al. 2012). The ITS region comprises ITS1 and ITS2, two highly variable spacers linked by the 5.8S gene. The ITS1 and ITS2 subregions are more commonly targeted (250–400 bases) compared with the entire ITS region being too long for metabarcoding with Illumina Miseq sequencing, which remains the most widely used HTS sequencing platform (Schoch et al. 2012; Tedersoo et al. 2015). Nevertheless, the ITS2 region generates less taxonomic bias than the ITS1 due to lower length variation with universal primer sites and has been proposed as a better alternative to ITS1 in the characterisation of fungal communities (Yang et al. 2018; Nilsson et al. 2019). Several primers have been designed to amplify ITS2 subregions exclusively belonging to the fungal kingdom (*i.e.* fungal-specific primers). However, most widely used fungal-specific primers also amplified DNA from other eukaryotes such as plants, for example, fITS7/ITS4, gITS7/ITS4, or 5.8Fun/ITS4-Fun (Ihrmark et al. 2012; Lindahl et al. 2013; Taylor et al. 2016; Koyama et al. 2019; Ricks et al. 2019; Botnen et al. 2020; Li et al. 2020; Chen et al. 2022).

When working on environmental samples, it is recommended to combine primers with degenerate positions (Ihrmark et al. 2012) and a lower annealing temperature (T_a_) than typically recommended (Bellemain et al. 2010) to improve the taxonomic coverage and facilitate amplification (Toju et al. 2012; Brown et al. 2018). Since amplification efficiency and specificity depend on the T_a_ (Yu and Wu 2011), T_a_ can be adjusted to limit the plant-fungal DNA co-amplification. Moreover, some rare ITS variants may be amplified only at a high T_a_ (Schmidt et al. 2013). However, increasing T_a_ can significantly reduce the yield of products (Rychlik et al. 1990) and the diversity recovered (Schmidt et al. 2013). Since the optimal T_a_ is often only determined based on electrophoresis agarose gel analysis (Terashima et al. 2002; Green et al. 2004; Banos et al. 2018), the specific taxa impacted by varying T_a_ are therefore not described.

Regardless of fungal primer pairs and PCR conditions, peptide nucleic acid (PNA) clamps have also been described as an efficient approach to discriminate fungal DNA from plant DNA (Giangacomo et al. 2021). These clamps are synthetic oligomers that bind to host-derived DNA and block its amplification during PCR (Lundberg et al. 2013; Fitzpatrick et al. 2018). PNA clamps were initially designed for and used in bacterial microbiome studies (Sakai and Ikenaga 2013; Fitzpatrick et al. 2018; Reigel et al. 2020; Kawasaki and Ryan 2021), and have subsequently been used for fungi (Cregger et al. 2018; Taerum et al. 2020). Most notably, Taerum et al. (2020) showed that PNA clamps reduced plant reads while increasing the number of fungal reads and associated diversity. Although some studies conducted on plant tissues have combined fungal-specific primers with ITS PNA clamps (*i.e*. ITS3/ITS4; 5.8S-Fun/ITS4-Fun; fITS7/ITS4; ITS1/ITS2) (Hamonts et al. 2018; Sun et al. 2020; Do et al. 2021; Lee and Hawkes 2021), to our knowledge, no studies have addressed which fungal taxa are affected when the ITS2 region is amplified. Moreover, it has been shown that blocking oligos can change fungal relative abundances and co-inhibit certain species with universal primers when sequencing arthropods (Piñol et al. 2015). Consequently, there is currently no consensus on the most appropriate PCR protocol for preferentially amplifying fungi over plants. Yet, in the study of fungal diversity within plant roots, the selection of primers, T_a_, and PNA clamps is pivotal for achieving this aim, and there is a notable absence of a systematic comparison regarding the impact and interplay of these technical elements.

This study aims to evaluate the combined effects of primer pairs, associated T_a_, and PNA clamps in determining the fungal community diversity and composition associated with plant roots. The plant used in this study is stinging nettle, *Urtica dioica* (L.). Although this plant grows in a wide range of habitats (Taylor 2009) and has raised scientific and commercial interests (Viotti et al. 2022), limited information is available on its associated microorganisms. Here, three primer pairs used in the literature were studied i) fITS7/ITS4, which is fungi-specific, ii) gITS7/ITS4, a universal primer pair amplifying fungi, plants, and protists, and iii) 5.8S-Fun/ITS4-Fun which is designed to be fungi-specific and to generate longer amplicons than the two other primer pairs (Taylor et al. 2016). The influence of a higher T_a_ (*i.e*. equal to the melting temperature of the primers) compared to the commonly used one was investigated for each primer pair, as well as the addition of PNA. Variations in relative abundance, OTU richness, and diversity indices of nettle root fungal communities were evaluated under various conditions of T_a_, PNA clamp use, and primer pair choice. The fungal taxa impacted by changes in PCR conditions were also identified. Finally, primer pairs and PNA clamps were evaluated using a synthetic mock community SynMock (Palmer et al. 2018) either alone or with varying ratios of plant to fungal DNA. The mock community was made from ITS-like fragments of different lengths and used as a control (Castaño et al. 2020) to assess both the influence of primers and PNA clamps, and to test the efficiency of plant DNA exclusion (Gohl et al. 2016; Pauvert et al. 2019).

## 2. Materials and methods

### 2.1. Sample collection and DNA extraction

In 2021, root samples from six adjacent stinging nettle (*U. dioica*) individuals were collected in Etobon (Bourgogne-Franche-Comté, France). Fine roots were first washed with tap water, followed by three baths of sterile distilled water, and an ultrasonic bath for 20 min to remove the soil attached to the roots. Samples were stored at −20 °C until analysis. Roots from an axenic *in vitro* stinging nettle from Rovaniemi (Luke, Finland) were also collected and processed identically. Total DNA was extracted from 50 mg of roots using the DNeasy PowerSoil kit (Qiagen, Venlo, The Netherlands) following the manufacturer’s instructions. Three extractions were performed on the same pool of roots and then combined for both environmental and *in vitro* roots. DNA from all samples was quantified with a Qubit® dsDNA HS Assay Kit (Invitrogen, Carlsbad, CA, USA). Additionally, a synthetic fungal mock community SynMock, (Palmer et al. 2018) comprising 12 ITS constructs was used as control, either alone or in combination with different ratios of *in vitro* stinging nettle to fungal mock DNA (*i.e*. 50:50 and 80:20).

### 2.2. DNA amplification and amplicon sequence library preparation

The fungal 5.8S and ITS2 region from nettle root samples, the SynMock community, and three negative controls were amplified with three Illumina-compatible primer pairs both with and without PNA clamps. The three primer pairs were fITS7/ITS4, gITS7/ITS4 (Ihrmark et al. 2012) (abbreviated thereafter fITS7 and gITS7, respectively) and 5.8S-Fun/ITS4-Fun (Taylor et al. 2016) (abbreviated thereafter 5.8S-Fun) (Table 1). Although, the clamps used in this study were designed to target the plant 5.8S nuclear RNA gene of *Populus* species (Cregger et al. 2018), but were also found to align with the ITS sequence of the herbaceous plant *Urtica dioica*, as extracted from the NCBI database. A two-step PCR was performed to prepare sequencing libraries. PCR1 was designed to achieve amplification of the target regions and to add Illumina Nextera transposase sequence to the amplicons. Both forward and reverse primers were amended with frameshift sequences (FS), after the Illumina Nextera transposase sequence, to improve sequence diversity and overall read quality (Caporaso et al. 2011). PCR1 was performed in triplicates for each sample. The reaction mixture (20 µL final volume) consisted of 4 µL of 5× Phusion Green High-Fidelity reaction buffer, 0.4 µL of 10 mmol dNTPs, 0.8 µL of each 10 μmol primers, 0.4 µL of Phusion High-Fidelity DNA Polymerase (ThermoFisher Scientific, Illkirch, France) and 10 ng of DNA template. The following cycling parameters were then used for amplification: An initial denaturation stage at 98 °C for 30 s; 35 cycles of denaturation at 98 °C for 15 s, annealing at 57 °C and 68 °C (fITS7 and gITS7) or 63 °C (5.8S-Fun) for 30 s, extension at 72 °C for 30 s, and a final extension at 72 °C for 10 min. For the PCR mix with PNA, 0.8 μL of 10 μmol PNA was added. PCR conditions were the same as before, except an additional step at 78 °C for 30 s was included before the primer annealing step. The quality of the PCR1 products was verified through electrophoresis on 1% agarose gels. Purification of PCR1 amplicons was achieved using Agencourt AMPure XP beads (Beckman Coulter, Chaska, MN, USA) at a bead-to-DNA ratio of 1:1. In PCR2 (Illumina dual indexing PCR), each cleaned PCR1 product within the same sample received a unique combination of forward and reverse primers (respectively, N7 and S5 Illumina dual index oligos). Samples were again cleaned afterwards, using AMPure XP magnetic beads, pooled in equimolar concentrations, and sequenced using 2 × 300 bp MiSeq v3 sequencing (Illumina Inc., San Diego, CA, USA).

**Table 1.** Amplicon size, fungal specificity, and the sequences of primers used.

Primers	Specificity for fungi	Sequences (5’–3’)	References
fIT7	98%	GTGARTCATCGAATCTTTG	Ihrmark et al. (2012)
gITS7	99%	GTGARTCATCGARTCTTTG	Ihrmark et al. (2012)
ITS4	98%	TCCTCCGCTTATTGATATGC	White et al. (1990))
*Amplicons size* (bp): 292			
5.8S-Fun	95%	AACTTTYRRCAAYGGATCWCT	Taylor et al. (2016)
ITS4-Fun	90%	AGCCTCCGCTTATTGATATGCTTAART	Taylor et al. (2016)
*Amplicons size* (bp): 440			
PNA	-	CGAGGGCACGTCTGCCTGG	Cregger et al. (2018)

### 2.3. Data processing and bioinformatic analysis

Three PCR controls were performed for each primer pair. Read counts obtained in PCR controls were subtracted from those of the samples. The “amptk” pipeline described in Palmer et al. (2018) was used to process the raw demultiplexed.fastq files. Following the removal of primers, sequences were trimmed to 250 bp, denoised using UNOISE3 (Edgar 2016) and clustered into operational taxonomic units (OTUs) at 97% similarity. Read counts in the OTU x sample matrix were adjusted to account for the tag-switch between samples with the amptk filter function “−t” that assesses the number of reads of mock community taxa present in environmental samples and removes all reads under this threshold from each sample. A hybrid algorithm that integrates results from USEARCH global alignment against the UNITE database v8 (Nilsson et al. 2018) and both UTAX and SINTAX Bayesian classifiers were used to assign taxonomy. The environmental dataset was rarefied to 49,007 sequences per sample, and the mock community dataset was rarefied to 138,195 sequences per sample.

### 2.4. Statistical analyses

All variables were checked for their homoscedasticity (Bartlett test) and normal distribution (Shapiro-Wilk test). Data that did not fit a normal distribution after transformation were analysed with non-parametrical tests. Differences in the number of fungal sequences from environmental samples and relative abundance of Viridiplantae kingdom among primer pairs and PCR conditions were assessed using a Kruskal-Wallis test followed by multiple comparisons from the “kruskal” function of the agricolae package (de Mendiburu 2019). Tukey’s HSD multiple comparison post-hoc analyses were used to assess the differences in the number of fungal sequences from the mock community. OTU richness and Shannon’s index were calculated with the vegan package (Oksanen et al. 2019) and compared between treatments using Kruskal-Wallis test followed by multiple comparisons. The effect of primer pair, PNA clamps, and a higher T_a_ was measured using linear mixed-effects models from the nlme package (Pinheiro et al. 2019). Fungal OTUs composition differences within the three primer pairs with or without PNA or higher T_a_ were plotted with a non-metric multi-dimensional scaling (NMDS) using the “meta MDS” function in the vegan package, based on the Bray-Curtis dissimilarity matrix. To determine the effects of the different primer pairs, PNA clamps, and a higher T_a_ potential on OTUs composition, we performed a permutational multivariate analysis of variance (PERMANOVA) based on Bray-Curtis dissimilarity using the “adonis” function from the vegan package. Bray-Curtis dissimilarity was calculated with the “vegdist” function from the vegan package between primer pairs with or without PNA or with a higher T_a_ for environmental samples and the mock community to measure the dissimilarity of fungal community composition. Statistical analyses and data visualisation were performed using R (R Core Team 2020) and were considered significant at *P* < 0.05.

## 3. Results

### 3.1. Mock community amplification

**Figure 1.**
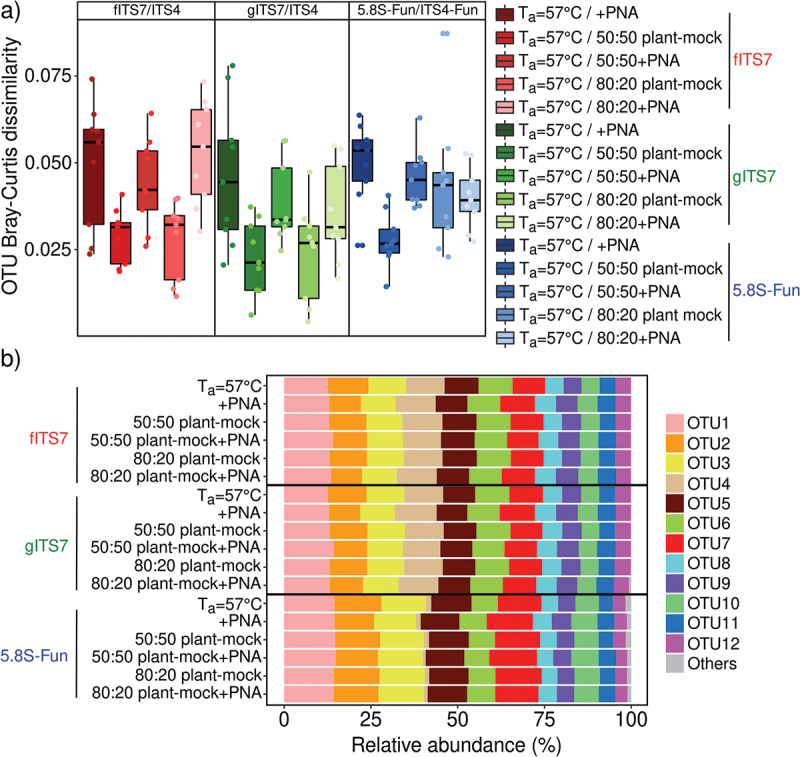
Variation in the SynMock community composition depending on the primer pair used (fITS7/ITS4 in red; gITS7/ITS4 in green and 5.8S-Fun/ITS4-fun in blue), the addition of PNA clamps (+PNA) and the ratio of plant-mock DNA (50:50 or 80:20). (a) OTU Bray-Curtis dissimilarity between classic amplification (T_a_ = 57 °C) and the various conditions tested. (b) Relative abundance of the 12 OTUs composing the SynMock.

We used a synthetic mock community to evaluate the potential bias of three primer pairs alone or combined with PNA clamps and to assess the impact of plant-fungal DNA co-amplification at different ratios. Sequencing of the mock community generated 11,322,947 fungal sequences before subsampling. The number of fungal sequences did not differ significantly between the three primer pairs and associated conditions (*i.e*. PNA clamps, different ratios of plant-fungal DNA) (Table 2). gITS7 primers added with plant DNA was the only treatment that generated plant reads (*i.e*. 464 and 2,052 Viridiplantae reads for 50:50 plant-mock DNA and 80:20 plant-mock DNA, respectively). The three primer pairs allowed the recovery of the 12 members of the mock community, and the OTU Bray-Curtis dissimilarity between the different treatments for each primer pair was low (<0.1) (Figure 1a). However, 5.8S-Fun exhibited differences in individual OTU relative abundances, ranging from less than 1% to 9.6% compared with the other primer pairs (Figure 1b). Moreover, 5.8S-Fun exhibited a higher number of additional OTUs (H_17,36_ = 39.7, *P* = 0.001), which exhibited a higher relative abundance (H_17,36_ = 44.3, *P* < 0.001). Experimentally reducing the plant-fungal DNA ratio did not impact the relative abundance of OTUs. On the contrary, adding PNA resulted in slight but significant changes in OTU relative abundance with the fITS7 and gITS7 primers (<2.5%; Figure 1b).

**Table 2.** Mean number of fungal sequences (±SE) obtained using the SynMock community for the three primer pairs (T_a_ = 57 °C) and the various combined treatments tested.

Primer pairs	T_a_ = 57 °C	+PNA	50:50 plant-mock	50:50 +PNA	80:20 plant-mock	80:20 +PNA
fITS7/ITS4	246,913 ± 11,955^a^	218,033 ± 17,439^ab^	236,290 ± 6,130^ab^	229,954 ± 14,493^ab^	204,413 ± 7,241^ab^	228,534 ± 42,884^ab^
gITS7/ITS4	205,999 ± 19,578^ab^	231,118 ± 6,776^ab^	242,530 ± 3,204^ab^	189,718 ± 3,601^ab^	217,261 ± 9,906^ab^	223,649 ± 14,853^ab^
5.8S-Fun/ITS4-Fun	169,416 ± 10,723^ab^	176,245 ± 13,371^ab^	204,221 ± 2,730^ab^	164,275 ± 16,281^ab^	200,470 ± 7,536^ab^	185,277 ± 10,225^b^

Different letters indicate significant differences between primer pairs and the tested conditions using a Kruskal-Wallis analysis followed by a post-hoc test using Fisher’s least significant difference, *P* < 0.05.

### 3.2. Plant-fungal DNA co-amplification in root samples

We amplified the same environmental samples with different PCR conditions (primer pairs, PNA clamps, a higher T_a_) to assess the impact on the level of plant-fungal DNA co-amplification. Illumina sequencing of environmental samples generated 4,167,783 fungal sequences and 346,249 Viridiplantae sequences after removing primers, short (<200 bp), low-quality sequences, and chimeras. The mean number of fungal sequences significantly differed between the three primer pairs and the associated treatments before subsampling (H_8,18_ = 20.2; *P* < 0.01). fITS7 generated a significant higher average number of fungal sequences than 5.8S-Fun (165,377 ± 7,786 and 99,339 ± 9,131 sequences respectively; Table S1) but did not differ significantly from gITS7 (114,656 ± 12,595 sequences). PNA clamps significantly increased the average number of fungal sequences for gITS7 (210,501 ± 14,611) and 5.8S-Fun (156,901 ± 3,197), while the higher T_a_ did not result in significant differences (Table S1).

**Figure 2.**
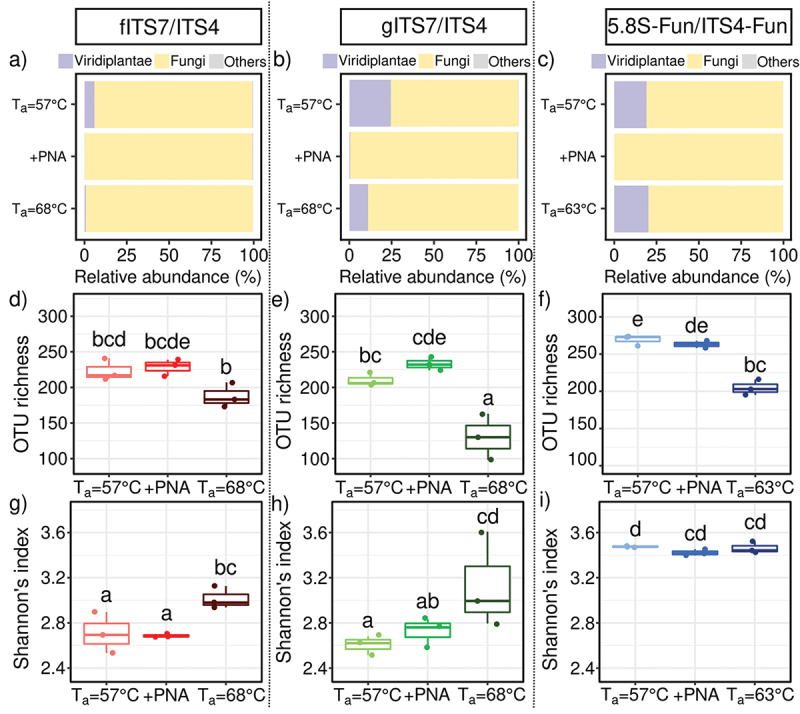
Abundance, richness and diversity of fungi in *Urtica dioica* roots for the three primer pairs tested fITS7/ITS4, gITS7/ITS4, and 5.8S-Fun/ITS4-fun (T_a_ = 57 °C), the addition of PNA clamps (+PNA) and the increase of T_a_ (T_a_ = 68 °C or 63 °C). (a–c) Relative abundance of reads of Viridiplantae, fungi, and other phyla (*i.e.* Amoebozoa, Choanoflagellozoa, Heterolobosa, Ichthyosporia, Metazoa, Protista, Rhizaria, rhodoplantae, Stramenopila, and NA); (d–f) Richness and (g–i) Shannon’s index. Boxes with the same letters did not differ significantly from each other using a Tukey-adjusted comparison and Kruskal-Wallis analysis followed by a post-hoc test using Fisher’s least significant difference, respectively, *P* < 0.05.

Primer pairs and T_a_ significantly impacted the relative abundance of Viridiplantae reads (H_8,18_ = 48.8; *P* < 0.001). gITS7 exhibited a four times higher relative abundance of Viridiplantae reads than fITS7 (Figure 2a,b), and it accounted for 19.2% of the relative abundance with 5.8S-Fun at both T_a_ = 57 °C and 63 °C (Figure 2c). Finally, PNA clamps completely inhibited plant DNA co-amplification with 5.8S-Fun, while fITS7 and gITS7 still recovered 0.1% and 0.5% of Viridiplantae sequences, respectively (Figure 2a–c). A higher T_a_ with fITS7 significantly divided it by seven (Figure 2a). We observed a similar trend but with a lower decrease for gITS7 with higher T_a_ (Figure 2b).

### 3.3. Fungal richness and diversity depending on PCR conditions and read depth

We compared the impact of different PCR conditions using environmental samples on the fungal richness and diversity to further assess the accuracy of primers. After subsampling, T_a_ and, at a lesser extent, the primer pairs had a significant impact on OTU richness (Figure 2d–f, F_8,18_ = 75.7; *P* < 0.001 and F_8,18_ = 21.0; *P* < 0.05, respectively), and Hill’s index (Figure S1, F_8,18_ = 51.0; *P* < 0.001 and F_8,18_ = 21.8; *P* < 0.001 respectively) compared to PNA clamps (*P* > 0.05). An elevated T_a_ significantly lowered the OTU richness with 5.8S-Fun and mostly with gITS7 for which the richness dropped by 40% (F_8,18_ = 22.2; *P* < 0.001). At T_a_ = 57 °C, 5.8S-Fun resulted in significantly more OTUs than fITS7 and gITS7 (Figure 2f). On the contrary, the primer pair had a greater impact on the Shannon index than T_a_ (F_8,18_ = 36.8; *P* < 0.001 and F_8,18_ = 12.7; *P* < 0.01, respectively). 5.8S-Fun resulted in a significantly higher Shannon index than the two other primer pairs (Figure 2g–i), and a higher T_a_ significantly increased it with fITS7 and gITS7 (H_8,18_ = 21.2; *P* < 0.01), while Hill’s index resulted in the exact opposite trend (Figure S1, H_8,18_ = 21.2; *P* < 0.001).

**Figure 3.**
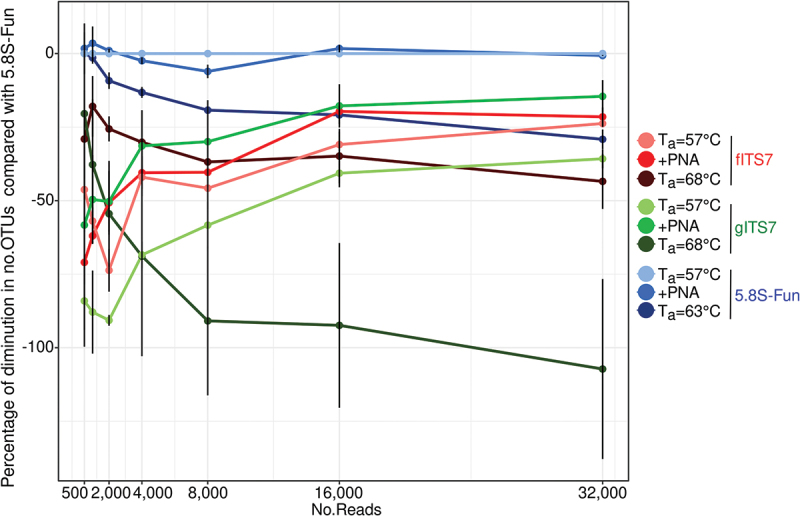
Percentage reduction in the number of OTUs for each primer pairs (fITS7/ITS4, gITS7/ITS4, and 5.8S-Fun/ITS4-fun) compared to the number of OTUs obtained with 5.8S-Fun, subsampled at various sequencing depths.

The percentage of diminution in the number of OTUs of the different treatments compared to 5.8S-Fun at T_a_ = 57 °C, which gave the highest OTU richness, was calculated to assess the quantification of fungal richness at different read depths. All the treatments exhibited a similar percentage of diminution compared to 5.8S-Fun between 8,000 and 32,000 read depths, with gITS7 at T_a_ = 68 °C that showed the highest decrease (Figure 3). Below a read depth of 2,000, fITS7 and gITS7 at T_a_ = 68 °C exhibited the lowest percentage of diminution, followed by fITS7 at T_a_ = 57 °C and gITS7 added with PNA clamps. PNA clamps significantly mitigated the decrease in OTUs by lowering the sequencing depth between 2,000 and 32,000 read depth with gITS7 (Figure 3).

### 3.4. Fungal taxonomic diversity and abundance

**Figure 4.**
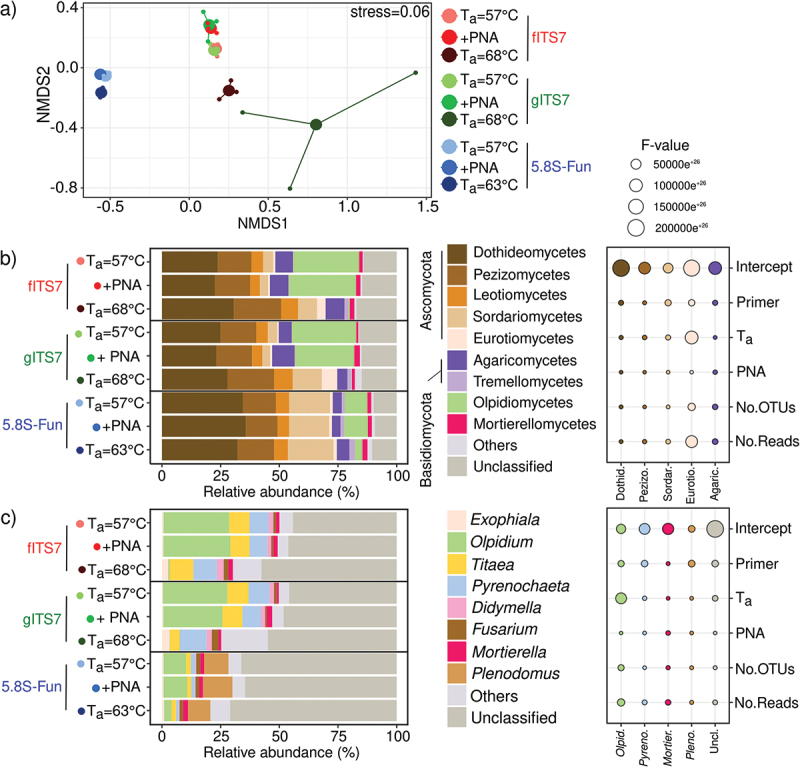
Fungal community composition in *Urtica dioica* roots depending on the primer pair used fITS7/ITS4, gITS7/ITS4, and 5.8S-Fun/ITS4-fun (T_a_ = 57 °C), the addition of PNA clamps (+PNA) and a higher T_a_ (T_a_ = 68 °C or 63 °C). (a) Non-metric multidimensional scaling (NMDS) analysis of the fungal communities, based on OTUs composition; (b) Relative abundance and *F*-value from linear mixed-effect models measuring the effect of primers, T_a_, PNA, the number of OTUs and the number of reads on the relative abundances of fungal classes and (c) Genera.

We studied the fungal diversity and abundance from the same environmental samples to identify the classes and genera impacted by different PCR conditions. PCR conditions notably affected fungal community structure (Figure 4a). PERMANOVA analysis showed that the primer pair was the most impacting factor (R^2^ = 0.75; *P* = 0.001), followed by T_a_ (R^2^ = 0.14; *P* = 0.001), while PNA clamps had no significant effect (R^2^ = 3.25e^−3^; *P* > 0.05). fITS7 and gITS7 were not significantly different in the relative abundances of the main fungal classes (Figure 4b). 5.8S-Fun, on the other hand, showed a significantly higher abundance of Dothideomycetes (+10%, F_8,18_ = 13.5; *P* < 0.001) and Sordariomycetes (+13%, H_8,18_ = 23.9; *P* < 0.01), a lower abundance of Olpidiomycetes (-18% H_8,18_ = 23.4; *P* < 0.01) (Figure 4b) and more OTUs assigned to Dothideomycetes (+20 OTUs, F_8,18_ = 32.9; *P* < 0.001), Eurotiomycetes (+5 OTUs, H_8,18_ = 19.3; *P* = 0.01) and Pezizomycetes (+4 OTUs, H_8,18_ = 20.8; *P* < 0.01) compared to the two other primers (Figure S2). The elevated T_a_ did not significantly impact the relative abundances of the main classes with 5.8S-Fun. On the contrary, a T_a_ = 68 °C with fITS7 and gITS7 significantly increased the relative abundances of Eurotiomycetes, Pezizomycetes, Sordariomycetes, and Tremellomycetes (H_8,18_ = 18.4; *P* < 0.05). The number of reads impacted these relative abundances more than the number of OTUs (Figure 4b). A higher T_a_ resulted in fewer OTUs assigned to the main classes but with a higher number of reads (Figure S2). PNA clamps only significantly increased by 2.5 times the relative abundance of Mortierellomycetes with gITS7 (F_8,18_ = 2.8; *P* < 0.05) but did not impact the number of reads. Also, four OTUs assigned to the Sordariomycetes were not recovered with fITS7 when PNA was added (Figures 4b, S2).

At the genus level, 5.8S-Fun had significant differences in relative abundances compared to fITS7 and gITS7, and allowed the recovery of the genus *Plenodomus* that accounted for 10.5% ± 0.4 of the relative abundance (Figure 4c, S2). Unclassified genera exhibited a higher relative abundance (+20% F_8,18_ = 38.1, *P* < 0.001), with 1.5 times more unassigned OTUs (F_8,18_ = 21.5, *P* < 0.001) and associated reads (F_8,18_ = 38.1, *p* < 0.001) (Figure 4c, S2). 5.8S-Fun also resulted in significant lower relative abundances of *Didymella* (H_8,18_ = 18.0, *P* < 0.05), *Titaea* (H_8,18_ = 18.6, *P* < 0.05), *Pyrenochaeta* (H_8,18_ = 23.4, *P* < 0.01) and *Olpidium* (H_8,18_ = 23.4; *P* < 0.01) (Figure 4c). A higher T_a_ mostly impacted the genus *Olpidium*, with a strong decrease in relative abundance (H_8,18_ = 24.0; *P* < 0.01) and a two- and eight-fold decrease in *Olpidium*-assigned OTUs with fITS7 and gITS7, respectively (H_8,18_ = 24.0; *P* < 0.01) (Figure 4, S2). On the contrary, a higher T_a_ significantly increased the relative abundances of unclassified genera and *Exophiala* with fITS7 and gITS7 (Figure 4c).

### 3.5. Primer pair specificity

**Figure 5.**
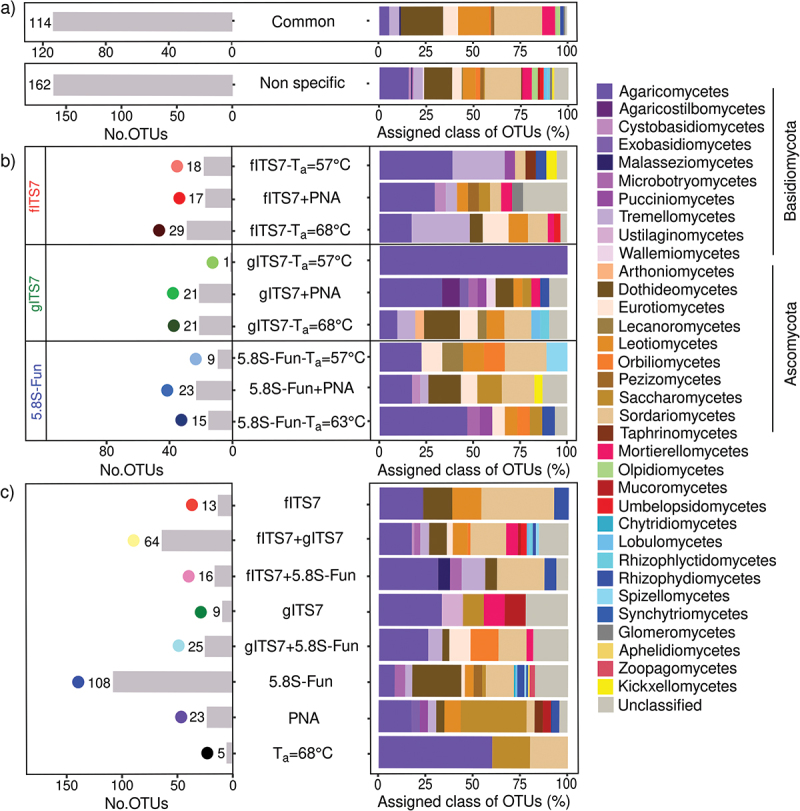
Representative classes and phyla of (a) non-specific or (b–c) specific OTUs of fungal community in *Urtica dioica* roots according to primer pairs (fITS7/ITS4, gITS7/ITS4, and 5.8S-Fun/ITS4-fun) and conditions used, as well as the number of OTUs corresponding. “Common” corresponds to the OTUs that were found in all PCR conditions with the three primer pairs. “Non-specific” corresponds to the OTUs that were found in at least one condition (T_a_ = 57 °C; +PNA or T_a_ = 68–63 °C) of PCR with the three primer pairs.

**Figure 6.**
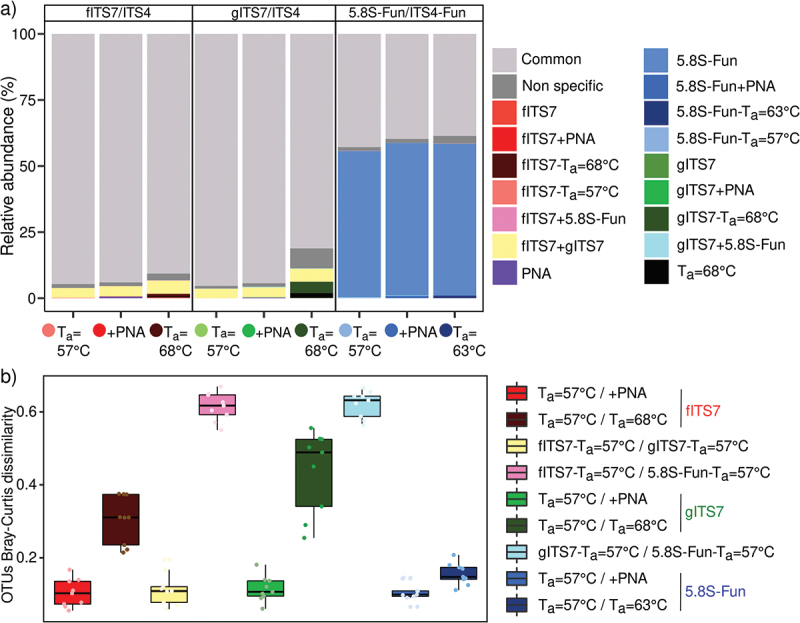
(a) Relative abundance of specific and non-specific OTUs for each primer pair used (fITS7/ITS4: red; gITS7/ITS4: green; 5.8S-Fun/ITS4-fun: blue), with the addition of PNA clamps (+PNA) and a higher annealing temperature (T_a_ = 68 °C or 63 °C); (b) OTU Bray-Curtis dissimilarity between the different primer pairs (T_a_ = 57 °C), with or without PNA clamps (+PNA), or with an annealing temperature of 68 °C or 63 °C.

We identified the OTUs recovered only in specific PCR conditions and their relative abundance to assess the taxonomic coverage of primers depending on the T_a_ and the use of PNA clamps. Sixteen percent of OTUs were shared to all PCR conditions tested (with the three primer pairs, with or without PNA clamps, with higher T_a_ = 68–63 °C, Figure 5a), and accounted for 95% of the relative abundance with the fITS7 and gITS7 primers but only for 43% with 5.8S-Fun (Figure 6a). 5.8S-Fun gave the highest number of specific OTUs, accounting for 55% of the relative abundance (Figures 5c,6a). On the contrary, fITS7 and gITS7 exhibited less specific OTUs that were poorly abundant (<4%). OTUs specific to 5.8S-Fun mainly belonged to the phylum Ascomycota while those specific to the two other primer pairs were mainly associated with the phylum Basidiomycota (Figure 5b,c). A higher T_a_ resulted in a few specific OTUs that accounted for less than 4% of the relative abundance (Figures 5b,c,6a). PNA clamps gave few, low abundant, and specific OTUs that covered a wide range of fungal classes from phyla Basidiomycota and Ascomycota (Figures 5c,6a).

**Figure 7.**
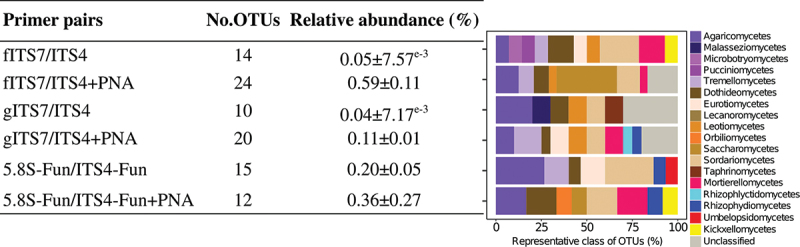
Number of OTUs found in at least two replicates, either without or with the addition of PNA clamps (+PNA), mean relative abundance in percentage ±SE for each primer pair (fITS7/ITS4, gITS7/ITS4, and 5.8S-Fun/ITS4-fun) and the percentage of represented fungal classes.

OTU Bray-Curtis dissimilarity confirmed that the main differences were between fITS7–5.8S-Fun, gITS7–5.8S-Fun, fITS7, and gITS7 at T_a_ = 57 °C and T_a_ = 68 °C (Figure 6b). Although the dissimilarity was low between T_a_ = 57 °C and PNA clamps, few OTUs were recovered only when they were added or not (Figure 7). These OTUs mainly represented by the phylum Ascomycota accounted for less than 1% of the relative abundance. Most OTUs could not be classified to either the genus (40%–75%) or the species (50%–83%) level.

## 4. Discussion

### 4.1. Primer pairs and plant-fungal DNA ratio affect plant DNA co-amplification

Since co-amplification of plant and fungal DNA can occur in studies focusing on the fungal microbiome associated with plant tissues, several methods have been proposed to alleviate this limitation (Harrison et al. 2021), notably by using fungal-specific primer pairs. In the present study focusing on plant roots, the fITS7 primer pair resulted in fewer plant reads than 5.8S-Fun, which has been described to exclude Viridiplantae sequences (Taylor et al. 2016). The non-specific fungal primer gITS7 yielded the highest number of plant reads (*i.e*. more than 20% of the relative abundance) in agreement with previously published results (Ihrmark et al. 2012). These results confirmed that different primer pairs led to varying levels of plant-fungal DNA co-amplification. This can be attributed to varying degrees of mismatch between the primer and the plant sequence, resulting in plant/fungal DNA co-amplification that varies depending on the plant species studied. Additionally, in our study, artificial modification of the plant-fungal DNA ratio to 50:50 and 80:20 before DNA amplification of the mock community resulted in no plant reads with the fungal-specific primers fITS7 and 5.8S-Fun with only a few plant reads detected using the more sensitive primer gITS7. Thus, the plant-fungal DNA ratio appears to substantially affect fungal sequence recovery from plant tissues under our experimental conditions. This result is in agreement with Lundberg et al. (2021) which reported a recovery of 100% of bacterial sequences from 24% of bacterial DNA. Variations in the plant-fungal DNA ratio within plant tissues might explain why Bodenhausen et al. (2019) observed up to 75% of plant sequences in *Petunia* roots, despite using a fungal-specific primer pair. Consequently, we suggest that plant roots with sparse fungal endophyte colonisation might be more susceptible to plant DNA contamination limitation.

### 4.2. Primer pairs affect fungal diversity and composition estimations independently of plant DNA co-amplification

In this study, among the three primer pairs tested on a plant mainly colonised by endophytic fungi and saprotrophs (Yung et al. 2021), the majority of sequences were fungal. After the removal of plant reads, the fITS7 primer pair yielded the highest number of fungal reads and 5.8S-Fun the lowest. Therefore, 5.8S-Fun does not appear to be the most suitable primer pair when the samples are plant tissues with a high plant:fungal DNA ratio (*e.g*. endophytes or pathogens with low colonisation levels), as it may result in low fungal sequencing depth. Additionally, we observed a higher number of additional OTUs, although they represented a low proportion in terms of relative abundance, that were not expected to be recovered from the mock community with 5.8S-Fun. This may suggest a higher index bleed with this primer pair or, more likely, potential chimeras, following the results of Li et al. (2020) that highlighted a higher non-specific amplification (*i.e*. chimera and mismatch) with 5.8S-Fun. However, 5.8S-Fun exhibited significantly higher OTU richness and Shannon’s index in environmental samples compared to fITS7 and gITS7. This finding is inconsistent with results based on soil samples (Li et al. 2020) and those from Ihrmark et al. (2012) that found more diverse amplicon communities with gITS7. Among the three primer pairs, members of Ascomycota were the most prevalent, following the trend of other studies (Li et al. 2020; Yung et al. 2021). The 5.8S-Fun primer pair yielded significantly more OTUs assigned to Ascomycota and Chytridiomycota (*e.g*. *Plenodomus*) than fITS7 and gITS7. This is consistent with the fact that fITS7 excludes some Ascomycota (Ihrmark et al. 2012). Furthermore, differentiating taxa within the phylum Chytridiomycota is challenging with variable markers such as the ITS region due to high genetic divergence and limited reference sequences available (Ishii et al. 2015; Tedersoo et al. 2015). Moreover, in a previous study, 5.8S-Fun yielded the lowest Basidiomycota abundance (Li et al. 2020). As the taxonomic specificity and coverage vary depending on the primer used (Baldrian et al. 2021), the impact of primer choice on the recovered diversity and richness of plant root-associated communities will depend on the fungal taxa harboured by the plant (*e.g*., dominance of Basidiomycota phylum over Ascomycota). Despite varying levels of plant DNA co-amplification, fITS7 and gITS7 resulted in comparable taxa coverage with only a few differences in the number of OTUs of some fungal classes that exhibited the same relative abundances. Overall, our results confirm that the primer pairs have a much more substantial impact on fungal diversity and composition assessment than plant-DNA contamination in our experimental conditions (*i.e*. high read depths).

### 4.3. Increased T_a_ reduces plant co-DNA amplification but alters the assessment of fungal diversity

Modifying T_a_ can contribute to limiting plant DNA co-amplification. In this study, increasing T_a_ significantly decreased plant-fungal DNA co-amplification with the fITS7 and gITS7 primers, but not with 5.8S-Fun. Although T_a_ can limit plant-fungal DNA co-amplification with some primers, it decreased the number of OTUs recovered, but not the sequencing depth. Schmidt et al. (2013) also described a decrease in the number of OTUs with a higher T_a_ when targeting the ITS1 region. Despite a lower OTU richness, raising the T_a_ allowed the recovery of specific OTUs. Shannon’s index, which was significantly higher with the fITS7 and gITS7 primer pairs, confirmed this observation. These specific OTUs, mainly assigned to Basidiomycota were found with the fITS7 and 5.8S-Fun primers. In addition, the relative abundance of Ascomycota increased despite a lower number of OTUs, suggesting that higher T_a_ decreases the recovery of Ascomycota-related fungi. That is contradictory with Larena et al. (1999), who used a high T_a_ (*i.e.* 62–64 °C) for the selective ascomycetous primer ITS4A. Additionally, increasing T_a_ highly impacted the relative abundance of the genus *Olpidium* with fITS7 and gITS7. Nevertheless, higher T_a_ with 5.8S-Fun had no impact on the diversity of rare OTUs nor the relative abundances of fungal classes or genera, suggesting that this primer pair is less sensitive to changes in T_a_. Our results indicate that while increasing T_a_ might help limit plant DNA contamination, it simultaneously reduces fungal diversity and alters fungal community composition.

### 4.4. PNA clamps efficiently exclude plant sequences without modifications in fungal diversity and composition

PNA clamps have been described in previous studies and were first used to inhibit the host DNA amplification in studies that aimed to characterise bacterial communities associated with plant tissues. They were later used for fungal studies (Lundberg et al. 2013). It was shown that PNA clamps are not universal and need to be designed based on the plant species (Fitzpatrick et al. 2018). Although the clamps used in this study were designed for *Populus* species, they efficiently excluded *U. dioica* sequences. In *Populus* roots, a primer mixture tagging the fungal ITS2 region, combined with 5.8S PNA, reduced plant reads from 98.4% to 1.2% (Cregger et al. 2018). In our study, the same concentration of ITS PNA clamp fully inhibited plant reads with 5.8S-Fun while few reads (<1%) were still recovered with the two other primer pairs. The efficiency of PNA clamps has been described as concentration-dependent (Belda et al. 2017), but our result suggests that the concentration of PNA should be adjusted depending on the primer pair used. Clamping in plant roots did lead to a greater sequencing recovery of other eukaryotes besides fungi, which still accounted for less than 1% in relative abundance, even with the non-fungal specific gITS7 primers. These results suggest that blocking plant DNA amplification did not result in an over-amplification of non-plant and non-fungal sequences.

PNA clamps increased the proportion of fungal reads, contrary to Borodušķe et al. (2023), but did not result in an over-amplification of fungal classes and genera. Also, PNA clamps did not impact OTU richness, Shannon index, or Hill index, and did not increase the recovery of rare microbial taxa, as described in Moccia et al. (2020). Instead, our results align with those of Fitzpatrick et al. (2018) on plant bacterial communities but differ from those of Steven et al. (2018) based on bee bacterial communities (Muñoz-Colmenero et al. 2020). PNA clamps slightly impacted the fungal composition during the amplification of the mock community. Clamping impacted several OTU relative abundances, with variations ranging from 2.5% to less than 1%, using fITS7 and gITS7 primers. This phenomenon was not observed in environmental samples except for Mortierellomycetes and gITS7 primers. PNA had little impact on the fungal composition compared with the unclamped samples. These results, in agreement with those of Reigel et al. (2020) on coral-associated bacteria, indicate a proportional DNA amplification of microbial communities with the increase in fungal sequences. Collectively, our results suggest that PNA clamps are useful for excluding plant sequences without introducing bias into the determination of fungal community composition.

### 4.5. PNA clamps improve sequencing depth and fungal richness determination for non-fungal specific primers at a low read depth

Although we demonstrated that PNA clamps significantly excluded plant contamination in our experiments, they did not enhance the observed fungal diversity. This might largely be explained by the fact that fungal reads accounted for at least 75% of total reads, even with gITS7, which is not fungal-specific. Consequently, when the plant sequence contamination is relatively low, and the sequencing depth is high, as it was the case in our experimental conditions, using PNA clamps seems unnecessary for estimating plant-associated fungal diversity and composition. Still, at artificially reduced sequencing depths, and particularly below 2,000 sequences per sample, we observed a substantial decrease in fungal richness for the non-fungal specific primers gITS7, which was alleviated by the use of PNA clamps, aligning it with the fungal-specific fITS7. When using non-specific primers, for instance, to ensure high taxonomic coverage (Op De Beeck et al. 2014), considering that rarefying OTU datasets at relatively low read depth as a normalisation approach is commonly employed (McKnight et al. 2019; Tedersoo et al. 2022), using PNA clamps appears as a suitable method to reduce plant DNA contamination and minimise the loss of fungal richness.

## 5. Conclusions

In this study, we tested the efficiency of primer pairs, T_a_, and PNA clamps to limit plant DNA contamination while assessing fungal diversity and composition in plant tissues. We highlight that using fungal-specific primer pairs to exclude plant sequences yielded mixed results, specifically that fungal-specific primer (5.8S-Fun) pairs led to similar levels of plant DNA contamination as the non-fungal specific primer (gITS7). Furthermore, increasing T_a_ reduced plant DNA contamination and improved the recovery of rare fungal taxa but had a substantial negative impact on the overall fungal richness. Finally, we demonstrated that PNA clamps efficiently suppressed plant amplification without introducing bias in fungal diversity and composition. The positive effects of PNA clamps on the fungal richness quantification were particularly significant at low read depth, and appear to be suitably combined with universal primers.

## Supplementary Material

Supplemental Material

## Data Availability

Raw sequence data for this project have been submitted to NCBI’s SRA archive under accession no. SUB12918264.
