# Supplementary figures and images for: Primer pairs, PCR conditions, and peptide nucleic acid clamps affect fungal diversity assessment from plant root tissues
Source: Mycology. 2024 Feb 4;15(2):255–71. doi: 10.1080/21501203.2023.2301003 (PMC11132971; doi:10.1080/21501203.2023.2301003)

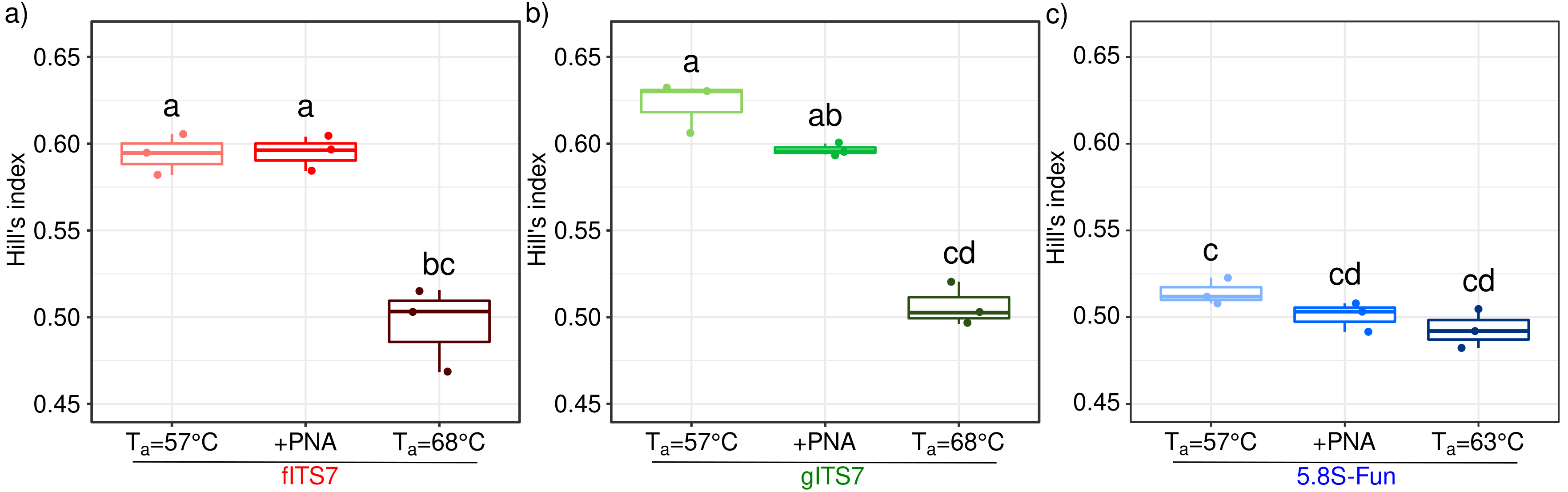

Supplement: Supplemental Material [file TMYC_A_2301003_SM9768.zip › FigS1.tiff]

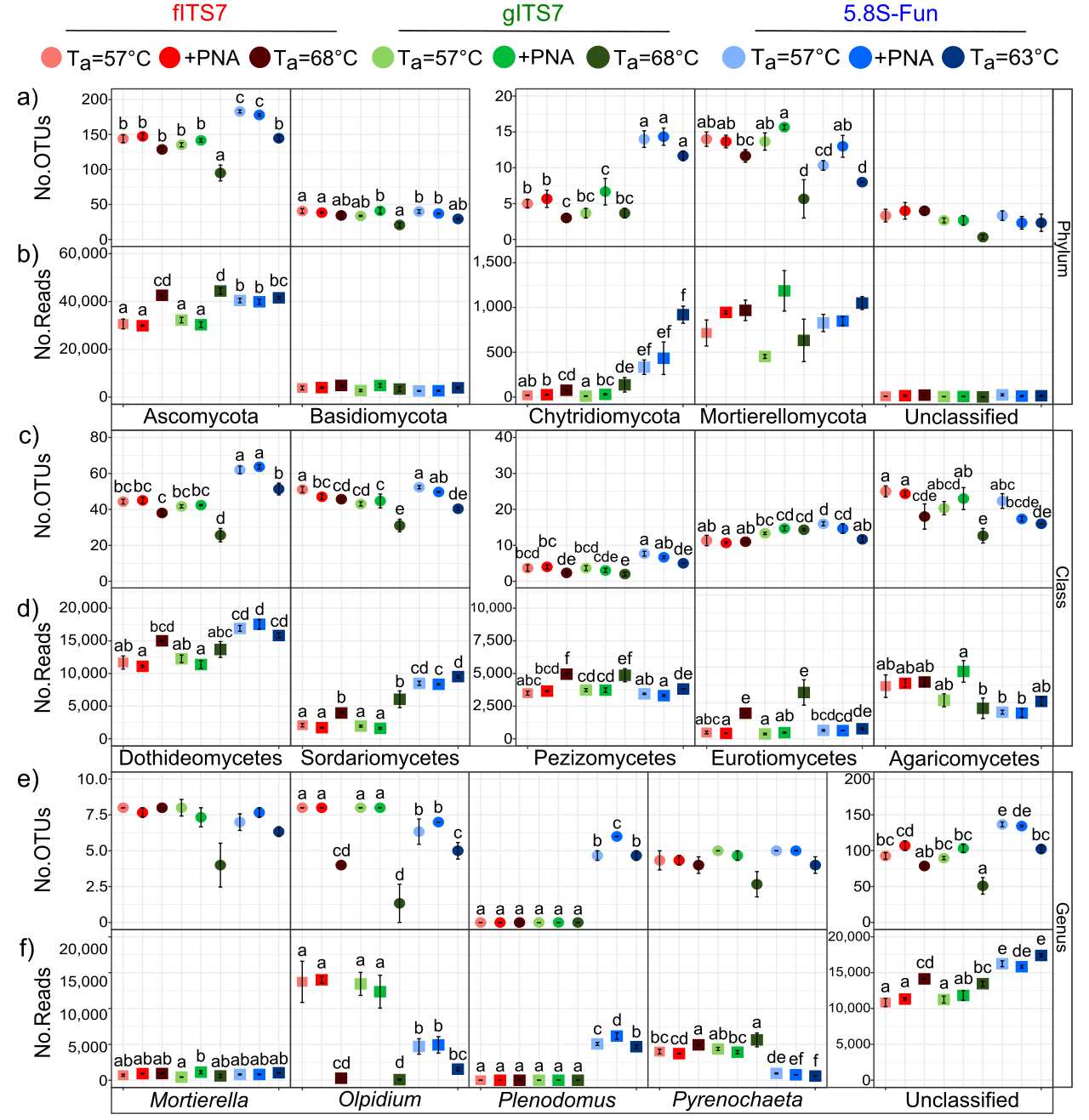

Supplement: Supplemental Material [file TMYC_A_2301003_SM9768.zip › FigS2.tiff]
